# Prognostic markers for colorectal cancer

**DOI:** 10.18632/oncotarget.26012

**Published:** 2018-09-04

**Authors:** Helen E.J. Tyrrell, David Kerr

**Affiliations:** David Kerr: Oxford University Hospitals Trust, Headington, Oxford, UK

**Keywords:** stage II colorectal cancer, adjuvant chemotherapy, ploidy, stroma-tumour fraction, prognosis

For patients with stage II colorectal cancer, adjuvant chemotherapy treatment can reduce the risk of cancer recurrence by 3-5%, but with a risk of significant side effects in 20% and a small risk of death [1]. Many patients will have been cured by surgery alone, and therefore better techniques are needed to identify patients at higher risk of recurrence, who are more likely to benefit from adjuvant treatment, or, indeed, those at lower risk of recurrence who have a higher chance of cure by surgery alone. We already know that the 12-15% of MMR deficient stage II colorectal cancer patients have a lower recurrence risk, and therefore in our centre, and many others, these patients are not routinely offered adjuvant chemotherapy [2, 3]. Mutant oncogenes such as RAS, MYC and P53 and the RNA signature, Oncotype Dx Colon, are relatively weakly prognostic and not in routine use [4]. A recent paper in Annals of Oncology [1] looks at two parameters, ploidy and stroma-tumour fraction, that can be analysed on ubiquitously available formalin fixed paraffin embedded (FFPE) tumour blocks, to analyse their impact on prognosis in stage II colorectal cancer.

Ploidy is a measure of cellular DNA content; diploidy being a normal amount, tetraploidy, double DNA content and aneuploidy >2n cellular DNA. Aneuploidy is a marker of chromosomal instability, which is an independently poor prognostic marker and one of the hallmarks of colorectal cancer. It is thought that these genetically unstable cells produce progeny with diverse genetic alterations, driving tumorigenesis. The chromosomal instability is thought to arise from damage to the cellular apparatus of mitosis in an early stage of tumour development [5]. In a high proportion of colorectal cancers, gains in quantity are seen for sections of chromosomes known to harbour oncogenes, such as chromosome 8q with the MYC transcription factor, and chromosome 13 with CDK8 and CDX2 [6].

The tumour environment is made up of both the epithelial tumour cells and the supporting stroma. The stroma consists of extracellular matrix and cells such as fibroblasts and immune infiltrative cells [1]. There is increasing evidence that the tumoural microenvironment is as important a determinant of outcome as the somatic tumoural mutational landscape, and a recent trial suggests tumours with a greater stromal component have a poorer prognosis [7]. This may be due to production of cytokines, growth factors and pro-angiogenic molecules by the stromal cells. It may also increase tumour cell invasiveness, possibly through induction of epithelial to mesenchymal transition. The CMS4 subtype of colorectal cancer is associated with a higher stromal component and more epithelial to mesenchymal transition [8].

The paper by Danielsen and coworkers demonstrated that both ploidy and tumour-stroma fraction were found to have significant prognostic impact in stage II patients, on multivariate analysis adjusted for T-stage, age and adjuvant treatment [1]. Primary colorectal cancer specimens were retrospectively analysed, from patients who’d taken part in the QUASAR 2 study, Gloucester cohort and Oslo University Hospital- Aker cohorts, which included 1029 stage II colorectal cancer patients. Ploidy was analysed by image cytometry and tumour-stroma fraction from histological images. The cut-off for defining low versus high stroma content was set at 50%.

Those patients with both diploid and low stroma tumours formed the best prognosis group, with 5 year cancer-specific survival (CSS) of 90% (95% CI: 87%-94%). Those with diploid and high stroma tumours, or those with non-diploid and low stroma tumours, formed an intermediate prognostic group, with 5 year CSS of 83% (95% CI: 79-86%). The worst prognosis group, with non-diploid and high stroma tumours had a 5 year CSS of 73% (95% CI: 65-82%). This gave a hazard ratio, compared to the low risk best prognosis tumours, of 1.8 (1.2-2.7) for intermediate risk tumours, and for high risk tumours of 2.9 (1.7-4.8). For these stage II patients, pathological T-stage and MSI status were also prognostic, but degree of tumour differentiation was not. It was found that the use of adjuvant chemotherapy did not change the hazard ratios associated with ploidy and stroma content, though for all stage II patients’ adjuvant treatment did reduce recurrence risk, with a hazard ratio of 0.44.

The paper also analysed the prognostic value of ploidy and tumour-stroma content on stage III colorectal cancers. The impact of these parameters was a lot less than that for the stage II cancers, with a 5 year CSS of 70% (95% CI: 64%-76%) for low-risk tumours, a 5 year CSS of 68% (95% CI: 64-71%) for intermediate risk tumours, and for high risk tumours a 5 year CSS of 58% (95% CI: 50-67%). This gave a hazard ratio for the high risk compared to the low risk groups of 1.5 (95% CI: 1.08-2.09). Therefore analysis of these parameters is unlikely to be clinically useful for this patient group, where the benefits of chemotherapy are greater, and other prognostic markers, such as T-stage and tumour differentiation, are more useful.

In clinical practise the results from this study could provide more accurate prognostic information for stage II colorectal cancer patients, who currently face difficult decisions about whether to proceed with adjuvant chemotherapy offered. It may also be used to support decisions about whether to offer adjuvant treatment to patients or simply to continue surgical follow-up. It may be possible to apply this robust and technically simple combination assay to other tumour types in which stroma and ploidy have been shown independently to be somewhat prognostic eg. prostate, breast and lung cancer.
